# Mechanical Complications of Proximal Femur Fractures Treated with Intramedullary Nailing: A Retrospective Study

**DOI:** 10.3390/medicina60050718

**Published:** 2024-04-26

**Authors:** Alvaro Lopez-Hualda, Esperanza Marin García-Cabrera, Marina Lobato-Perez, Javier Martinez-Martin, Giacomo Rossettini, Massimiliano Leigheb, Jorge Hugo Villafañe

**Affiliations:** 1 Orthopedic Surgery and Traumatology Service, Hospital Universitario Fundación Alcorcon, 28922 Alcorcón, Spain; alvaro.lopez3@universidadeuropea.es (A.L.-H.); emaring@salud.madrid.org (E.M.G.-C.); mlobatoper@gmail.com (M.L.-P.); javiermm@salud.madrid.org (J.M.-M.); 2 Department of Physiotherapy, Faculty of Sport Sciences, Universidad Europea de Madrid, 28670 Villaviciosa de Odón, Spain; 3 Musculoskeletal Pain and Motor Control Research Group, Faculty of Sport Sciences, Universidad Europea de Madrid, 28670 Villaviciosa de Odón, Spain; 4 Musculoskeletal Pain and Motor Control Research Group, Faculty of Health Sciences, Universidad Europea de Canarias, Tenerife, 38300 Canary Islands, Spain; 5 Department of Human Neurosciences, University of Roma “Sapienza Roma”, 00184 Rome, Italy; 6 School of Physiotherapy, University of Verona, 37129 Verona, Italy; 7 San Gaudenzio Clinic (Monza Polyclinic Group), 28100 Novara, Italy; massimiliano.leigheb@gmail.com

**Keywords:** hip, fracture, intramedullary nailing, mechanical complications, TFNA implant, risk factors

## Abstract

*Background and Objectives*: This retrospective cohort study analyzes mechanical complications in hip fracture surgery using the Trochanteric Fixation Nail-Advanced (TFNA) implant. It investigates the correlation of these complications with demographic, intraoperative, and radiological factors, aiming to identify associated risk factors and suggest improvements in clinical surveillance and treatment strategies. *Materials and Methods*: We enrolled 253 patients diagnosed with pertrochanteric hip fractures treated between 2017 and 2021, with 126 meeting the criteria for a minimum 6-month follow-up. Data on demographics, American Anesthesia Association Classification (ASA), comorbidities, AO/OTA [AO (Arbeitsgemeinschaft für Osteosynthesefragen)/OTA (Orthopedic Trauma Association)] fracture classification, procedural details, and time to failure were collected. Radiographs were evaluated for reduction quality, the tip–apex distance (TAD), progressive varus deviation, and identification of mechanical complications. Statistical analysis was performed using SPSS software. *Results*: The predominant AO/OTA fracture classification was 31A2 in 67 cases (52.7%). Reduction quality was deemed good or acceptable in 123 cases (97.6%). The mean time to failure was 4.5 months (range: 2.2–6). The average TAD was 18 mm (range: 1.2–36), with a mean progressive varus deviation of 2.44° (range: 1.30–4.14). A good or acceptable reduction quality was observed in 97.6% of cases. Mechanical complications occurred in 21.4% of patients, with significant associations found with the lateral cortex fracture, use of a TFNA implant with a 130° angle, open reduction, and absence of prior osteoporosis treatment. *Conclusions*: The study provides insights into mechanical complications in proximal femur fractures treated with the TFNA nail, emphasizing the need for enhanced clinical and radiographic surveillance, especially in patients without osteoporosis treatment. Our findings support the necessity for further clinical studies comparing these outcomes with other implant designs and underscore the importance of personalized treatment strategies to reduce complication rates.

## 1. Introduction

The increasing incidence of proximal femoral fractures, both intracapsular and extracapsular, presents a significant challenge in the orthopedic trauma arena, particularly among the elderly population [1]. These fractures often result from low-impact incidents, such as falls from a standing position, which are common among older adults [2]. Contributing factors like osteoporosis and diminished stability substantially increase susceptibility to these fractures [3]. Studies have shown a correlation between advancing age and an increased fracture risk, with women being at a higher risk [2]. This trend reflects demographic shifts towards longer life expectancies and heightened vulnerability to osteoporosis among aging populations [2].

Despite advancements in intramedullary nailing techniques and implant design, mechanical complications associated with the treatment of these fractures remain a significant issue [1]. These complications, including implant failure, non-union, malalignment, cut-out, and other mechanical challenges, can dramatically affect post-surgical recovery and the quality of life of patients, thus presenting a considerable challenge for orthopedic surgeons and healthcare systems [2]. The rarity of mechanical failure in intramedullary nails, while generally reassuring, belies the potential severity of such events. Given the high volume of proximal femur fracture cases treated globally, even infrequent occurrences of mechanical complications can significantly impact patient outcomes and impose a substantial clinical and economic burden [3].

Decreased bone density, frequently associated with conditions like osteoporosis, renders the proximal femur vulnerable to fractures by reducing its ability to withstand mechanical stress. Intra-articular fractures present specific difficulties as they may disrupt blood flow to the femoral head, increasing the likelihood of avascular necrosis [4]. Clinicians employ classification systems like Garden and Pauwels classifications for femoral neck fractures to determine the best treatment strategies [5]. These categorizations aid in assessing the degree of the fracture displacement and angle, which are important factors in determining the probability of complications like nonunion or avascular necrosis [5].

Proximal femur fractures have an important economic and societal effect [6]. The economic and societal impact of proximal femur fractures is substantial, encompassing costs related to surgical interventions, hospitalization, postoperative care, therapy, and diminished independence [7,8]. These fractures also have significant effects on both the rates of mortality and morbidity [9]. The fluctuation in occurrence throughout different seasons suggests environmental hazards like slippery conditions during winter as contributing factors [1].

Orthopedic interventions have the goal of reinstating functionality and lessening adverse outcomes [10]. The treatment alternatives span from non-surgical management, which is seldom employed because of the elevated likelihood of complications, to surgical interventions [11]. The decision regarding surgical intervention hinges on several factors including the nature of the fracture, the patient’s age, existing health conditions, and the overall functional capacity [11]. Internal fixation is frequently favored for intracapsular fractures that are not displaced in younger patients, whereas arthroplasty might be chosen for older patients with displaced fractures to minimize the risk of lack of healing and avascular necrosis [12].

Intramedullary nailing is a popular surgical approach for treating proximal femoral fractures, especially subtrochanteric fractures [4]. Its popularity is due to its biomechanical advantages in distributing loads and the minimally invasive nature that protects the surrounding soft tissue [1]. This method enhances rapid recovery and reduces the risk of complications, showcasing how advanced surgical procedures can successfully manage complex fracture scenarios [1].

Proximal femur fractures, commonly treated with intramedullary nailing, can lead to various mechanical complications, which can significantly impact the outcomes and quality of life of affected patients. These complications encompass a spectrum of issues, including implant failure, non-union, malalignment, cutout, and other mechanical challenges [13].

It is worth noting that the mechanical failure of intramedullary nails is generally considered a rare occurrence [14]. Intramedullary nailing techniques have evolved over the years, and modern implant designs, such as the Trochanteric Fixation Nail-Advanced (TFNA)™ implant by DePuy Synthes, have been engineered with a focus on stability and durability [15]. The TFNA was introduced to the global market in 2015 and is made from a titanium–molybdenum alloy (TiMo). TiMo has also been shown to have a lower elastic modulus compared with other nail designs. As an innovation, the nail includes a lateral relief cut, through which the lateral portion of the proximal nail is progressively withdrawn from proximal to distal, leaving a flattened rather than cylindrical shape, to preserve bone. However, this has generated a reduction in the thickness of the proximal wall of the nail. As a result, the overall incidence of mechanical failure in intramedullary nailing procedures for proximal femur fractures is relatively low. However, when such failures do occur, they can have profound consequences for patients, often necessitating revision surgery and posing a considerable clinical and economic burden.

While the rarity of mechanical failure in intramedullary nails is reassuring, it is essential to recognize that even infrequent occurrences can have a significant impact given the high volume of proximal femur fracture cases globally. Understanding the factors contributing to mechanical complications, including the rare instances of implant failure, remains crucial for further improving the safety and efficacy of surgical interventions in these fractures. This study is motivated by the observation that, despite an overall reduction in the incidence of these complications due to the evolution of techniques and implants, their occurrence continues to present significant challenges. Therefore, there is a need for further investigation into these complications to identify risk factors and establish correlations with various demographic, intraoperative, and radiological variables [16]. This retrospective cohort study aims to delve into the broader landscape of the mechanical complications of hip fractures treated with intramedullary nailing, including both common and rare events, to provide a comprehensive overview of the challenges encountered in clinical practice.

## 2. Materials and Methods

### 2.1. Variable

**Figure 1:**
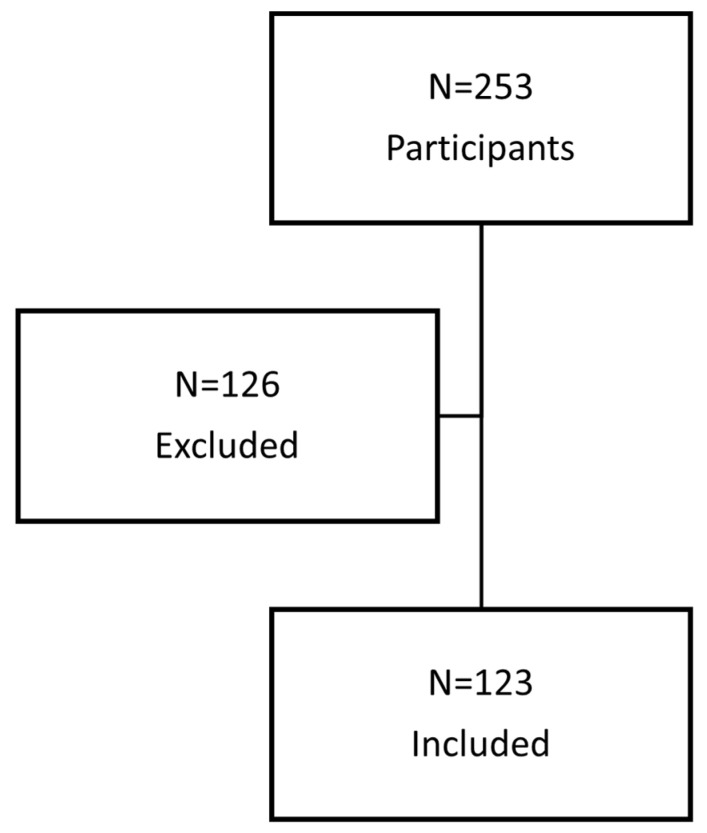
Patient Selection Process for the Study of Pertrochanteric Hip Fractures Treated with TFNA™ Implant.

This retrospective observational study targeted patients afflicted with pertrochanteric hip fractures who underwent intramedullary nailing employing a TFNA™ implant within the timeframe spanning 2017 to 2021. A mandatory follow-up period of at least 6 months, encompassing both clinical and radiological assessments, was instituted. Exclusion criteria encompassed individuals with pathologic or bilateral fractures, metastatic cancer, or those lacking pivotal clinical or radiological data. Out of the initial cohort of 253 patients subjected to screening, only 126 fulfilled the predetermined inclusion criteria (Figure 1).

Ethical endorsement for this investigation was secured from the Institutional Ethics Committee (TFGM2227).

### 2.2. Clinical Measurements

Patient records underwent meticulous scrutiny to extract demographic particulars, co-morbidities, body mass index (BMI), smoking status, osteoporosis treatment, American Anesthesia Association Classification (ASA), and preoperative functional autonomy for basic activities of daily living. Intraoperative variables were collected, including the need for open or closed reduction, implant characteristics (length, diameter, and angulation), and intraoperative complications, among others.

Subsequently, an exhaustive radiological analysis was performed, assessing AO/OTA fracture classification, lateral cortex fracture, Medial cortex contact AP view quality of the reduction according to Chang’s criteria, position of the blade according to the Clevaland classification, tip–apex distance, and progressive varus deviation and identification of mechanical complications. Mechanical complications encompassed cut-out, implant breakage, cut-through, back-out, and peri-implant fracture.

### 2.3. Surgical Procedure

All surgical interventions were conducted under the aegis of regional anesthesia with patients placed in a supine orientation upon a specialized fracture table. Closed reduction endeavors were undertaken employing traction and rotational maneuvers guided by fluoroscopy. In cases of intricate fractures, open reduction was deemed necessary prior to intramedullary nailing. The precision placement of the blade tip during fixation aimed to center it within or slightly below the femoral head on the anteroposterior (AP) projection and precisely at the midpoint on the lateral projection.

### 2.4. Postoperative Management

Postoperative care protocols encompassed early mobilization, full weight-bearing, and prophylactic antithrombotic measures entailing daily subcutaneous administration of 40 mg Enoxaparin sodium for a duration of 30 days. Comprehensive physiotherapeutic interventions were tailored towards functional restoration, including transfers, ambulation, seated mobility, equilibrium maintenance, and fall risk mitigation strategies.

Mechanical complications were identified in routine check-ups in consultation or when the patient went to the emergency room due to pain and inability to walk. All patients diagnosed with mechanical complications were admitted for preoperative study through blood analysis and radiological study with CT. The intervention was performed as soon as the patient’s general condition permitted after evaluation by the anesthesia service.

### 2.5. Data Measurement

Patients underwent systematic follow-up evaluations at 1, 6, and 12 months postoperatively at outpatient clinics. Radiological assessments, comprising AP and axial imaging, were conducted by seasoned radiologists. The criteria for bone union included the absence of clinical pain during hip mobilization and weight-bearing, corroborated by radiographic evidence depicting bridging of at least 3 cortices across two distinct views. Preoperative radiographs were scrutinized to categorize fracture patterns based on the revised AO/OTA [AO (Arbeitsgemeinschaft für Osteosynthesefragen)/OTA (Orthopedic Trauma Association)] classification system. Assessment of reduction quality, lamina positioning, tip–apex distance (TAD), progressive varus deviation, and identification of mechanical complications was carried out by a panel of at least two orthopedic specialists. TAD, a pivotal metric, was computed as the summation of distances from the screw tip to the femoral head center on AP and axial radiographs, with a target threshold set at less than 20 mm.

### 2.6. Statistics

The statistical analysis was conducted using SPSS version 28.0 software (SPSS Inc., Chicago, IL, USA). Descriptive statistics, comprising mean and standard deviation, were computed for all patients. Fischer’s exact test and the chi-square test were employed to explore associations and dependencies among the variables, whereas ANOVA was utilized to assess changes in variance. A significance level of *p* < 0.05 was deemed indicative of statistical significance.

## 3. Results

### 3.1. Clinical Characteristics of the Participants

We observed 253 cases of intramedullary fixations utilizing the TFNA™ implant during the period spanning from January 2017 to December 2021. However, the final analysis focused on 126 patients who satisfied the inclusion criteria and completed a 6-month follow-up.

### 3.2. Descriptive Data

Demographically, the patients’ cohort exhibited an average age of 81 years (range: 65–96) and an average BMI of 26.42 (range: 15.16–41.41). A significant majority were female (76.2%), non-smokers (83.3%), and had not undergone osteoporosis treatment (77.8%). The ASA classification revealed a distribution of 0.8% of type 1, 52.4% of type 2, 41.3% of type 3, and 5.6% of type 4. Among the patients, 95 had a history of hypertension (75.4%), 42 diabetes (33.3%), and 74 dyslipidemia (58.7%), as shown in Table 1.

All subjects presented with pertrochanteric femur fractures, primarily categorized as type 31A2 (57.2%), with the remainder comprising 31A1 (24.6%) and 31A3 (18.3%). Lateral cortex extension was noted in 37.3% of cases. Right-hip fractures were observed in 56.3% of patients, while left-hip fractures were found in 43.7%, as shown in Table 1.

The TFNA™ implant, equipped with a blade, was uniformly employed for all patients, with variations in the nail diameter and length. Cephalic screw angulation predominantly adhered to 130° (80.2%), whereas 125° was utilized in the remaining cases (19.8%). The majority of nails were secured with static distal locking (80%), while the remainder incorporated dynamic locking with a single screw.

After the evaluation of the reduction quality, based on Chang’s criteria, 46 cases were categorized as good, 75 cases as acceptable, and 4 cases as poor. The average TAD measured 18 mm (range: 1–36 mm), and an average progressive varus deviation of 2.44° was observed (range: 1.30–4.14), as shown in Table 2.

### 3.3. Outcome Data

The mean interval between the index surgery and the manifestation of a mechanical complication was 4.5 months (range: 2.2–6). Mechanical complications occurred in 21.4% of cases, with some patients experiencing multiple simultaneous complications.

Major mechanical complications encompassed cut-out, implant breakage, and cut-through, characterized by their substantial clinical impact and the necessitation of intricate revision surgeries. Conversely, back-out was considered a minor complication due to its lower propensity for requiring revision surgery and its generally more straightforward management.

Of the major complications, implant breakage occurred in four patients, cut-out in eight, and cut-through in four. Minor back-out complications were observed in 22 patients, as shown in Table 3.

The treatment of implant breakage primarily involved re-nailing with a longer, larger-diameter nail, often accompanied by bone autograft. Material extraction was performed in instances of cut-out, cut-through, and back-out. Cut-out cases underwent partial hip replacement, while the remaining cases were managed conservatively due to the high comorbidity of the patients. In particular, we performed revision nailing in four cases, arthroplasty in three cases, material extraction in five cases, and nonoperative management in four cases.

### 3.4. Main Results

Mechanical complications exhibited significant associations with specific factors, notably, the presence of lateral cortex fractures, the utilization of a TFNA™ implant with a 130° angulation, the open reduction of the fracture, and the absence of prior osteoporosis treatment. Particularly noteworthy was the association between the absence of prior osteoporosis treatment and the development of mechanical complications (*p* = 0.051), with such complications occurring in 14.3% of patients who had received osteoporosis treatment and in 33.3% of those who had not.

Fractures involving lateral cortex rupture and the requirement for open reduction signified highly unstable and complex fractures, with 39.1% of patients experiencing mechanical complications following open reduction and 31.9% of fractures with an observed lateral cortical rupture developing complications.

Finally, it was observed that implants featuring a 130° angulation were associated with a heightened risk of complications, with all patients who experienced mechanical complications utilizing this type of implant.

## 4. Discussion

Our study offers a unique and comprehensive examination of the mechanical complications associated with the use of the Trochanteric Fixation Nail-Advanced (TFNA™) implant in managing proximal femur fractures. Unlike previous studies that have broadly addressed hip fracture management, our research specifically explores the correlation between mechanical complications and various influential factors such as patient demographics, surgical techniques, and specific characteristics of the implant. This detailed analysis not only provides a deeper understanding of the risk factors linked to mechanical complications, but also marks a significant enhancement to the literature on this topic.

Before our investigation, there was a noticeable deficiency in the literature regarding the specific risk factors leading to mechanical complications in proximal femur fractures treated with the TFNA system. We have addressed this gap by identifying critical factors that contribute to such complications. These include the presence of lateral cortex fractures, the employment of a TFNA implant with a 130° angle, the necessity of open reduction during surgery, and the absence of prior osteoporosis treatment. By spotlighting these factors, our study facilitates more targeted patient management and surgical planning, which could potentially decrease the frequency of mechanical complications.

### 4.1. Main Findings

The primary objective of our present investigation was to assess the outcomes of hip fracture surgery using the TFNA™ implant and explore potential correlations with a range of factors, including patient demographics, intraoperative variables, and radiological assessments. Our findings indicate that the utilization of the TFNA™ system is characterized by high-quality reduction, precise implant positioning, and a 20% of postoperative complications. This study provides valuable insights into the mechanical complications related to proximal femur fracture surgery employing the TFNA™ implant.

### 4.2. Comparison with Other Evidence

A noteworthy observation in our study was the significant representation of female subjects within our patient cohort, a trend consistent with findings from previous investigations. While our statistical analysis did not establish a significant correlation between gender and the occurrence of post-surgical complications when employing the TFNA™ system, it does emphasize the inherent vulnerability of women to proximal femur fractures and their potential complications.

Additionally, our study unveiled a significant association between the absence of prior osteoporosis treatment and the development of mechanical complications. Patients who had not received osteoporosis treatment exhibited a higher rate of complications (33.3%) compared to those who had received such treatment (14.3%). This finding aligns with the existing literature, which reports an overall mechanical complication rate of up to 20% in similar studies [17,18,19].

It is important to note that prior research has employed varying definitions of “failure”, encompassing any circumstances necessitating additional surgical interventions, as exemplified by Matre et al. [20]. In contrast, our study meticulously categorized and differentiated between different complication scenarios, designating them as primary outcome parameters. Cases like implant removals in young patients without reported issues were excluded from the classification of complications within the scope of this investigation. Additionally, cases of infection, contributing to mechanical failure, were not included [21,22,23,24,25,26,27].

The study by López-Hualda et al. [28] conducted a comparative analysis of two fixation systems, DHS and TFNA™, for the treatment of intertrochanteric hip fractures, with a primary focus on clinical outcomes, complications, and mortality. Their results revealed that the TFNA™ group exhibited a significantly higher success rate in achieving a full weight-bearing capacity at the time of hospital discharge, particularly in cases involving unstable fractures. Furthermore, the study emphasized the association between surgical delay and increased mortality among patients with these fractures, underscoring the critical importance of prompt surgical intervention in the management of intertrochanteric hip fractures. These findings carry substantial implications for selecting the appropriate fixation system and the timely management of intertrochanteric hip fractures [29].

The socioeconomic consequences of proximal femur fractures are multifaceted. These fractures result in direct healthcare costs related to hospitalization, surgical procedures, postoperative care, rehabilitation, and ongoing patient management. Additionally, there are indirect financial implications, including reduced productivity, decreased self-reliance, increased dependence on assistance, and the potential need for long-term care. The overall economic impact is substantial, affecting not only individuals, but also placing a burden on healthcare systems, insurers, and society as a whole. Over recent decades, this medical condition has led to a growing demand for resources. The optimal approach to ensure early weight-bearing and mobility while employing minimally invasive surgical techniques remains a subject of ongoing debate and controversy. As the aging population continues to grow, the socioeconomic impact of osteoporotic proximal femur fractures becomes increasingly significant. A significant proportion of these patients, more than 30%, are unable to return to their pre-fracture place of residence. Despite ongoing advancements in implant design, the reported rates of implant-related failures still fall within the range of 2.0% to 16.5% [30]. The majority of these failures result from poor underlying bone quality or the improper positioning of the cephalic implant, which can lead to complications such as cut-out and varus collapse. Such complications often necessitate further surgical interventions, which can be particularly challenging in an already vulnerable population of hip fracture patients [31]. Both fracture reduction and implant positioning are well-established factors in preventing these complications. The use of metrics like the TAD and the more recently introduced calcar-related TAD has highlighted the importance of avoiding the eccentric (anterior, posterior, or superior) placement of the head element (HE) to mitigate the risk of mechanical failures. However, real-world clinical scenarios may not always permit ideal implant positioning, and reports on complications following hip fracture surgeries indicate that surgeons may occasionally accept an eccentric HE placement when necessary [8].

### 4.3. Limitations

This retrospective cohort study encounters several limitations that are crucial for a comprehensive interpretation of its findings. The retrospective design inherently introduces potential selection bias and risks of data incompleteness. Despite incorporating a considerable patient cohort, the exclusion of cases due to unavailable clinical or radiological information might have occurred, possibly affecting the study’s comprehensiveness and outcomes. A notable limitation arises from the radiological assessments employed. While established criteria were utilized for evaluating reduction quality and other radiographic parameters, the interpretation of these radiographs is subject to inter-observer variability. This variability introduces an element of subjectivity, which could impact the consistency and reliability of the measurements. Moreover, the identification of significant risk factors for mechanical complications in patients treated with the TFNA™ intramedullary nail, although insightful, is inherently limited by the observational nature of the study. This aspect underscores the potential biases, including those related to patient selection, which might not fully represent the broader population undergoing hip fracture surgery with the TFNA implant. Such biases are critical to acknowledge as they could influence the study’s observed outcomes and their generalizability. This study’s sample size, while robust, may not possess the statistical power necessary to detect smaller effect sizes or to conduct detailed sub-group analyses. Consequently, certain potentially significant correlations between patients or surgical characteristics and mechanical complications might remain unidentified. The statistical methodologies employed also present their limitations. These include assumptions of linear relationships in regression analyses where the actual relationships might be non-linear, as well as the potential for multicollinearity when multiple predictors are included in the models. These limitations could compromise the precision of estimated associations between risk factors and mechanical complications. Additionally, the reliance on retrospective data extracted from medical records and radiographs carries the risk of measurement bias. The quality of radiographs, the variability in their assessment by different observers, and the accuracy and completeness of medical records could all influence the statistical outcomes.

### 4.4. Implications for Clinical Practice

Our research has shown that there are significant links between mechanical complications and certain factors, such as lateral cortex fractures, the use of TFNA implants with a specific angle, the need for open reduction, and the absence of prior osteoporosis treatment. These findings highlight key areas for improvement in clinical practice. It is important for healthcare professionals to identify patients who are at high risk for complications before surgery and to create personalized management strategies accordingly. This may involve a more thorough preoperative planning process, where potential risk factors are carefully assessed and reduced through tailored surgical approaches and postoperative care protocols.

This study emphasizes the importance of integrating osteoporosis care into the treatment regimen for patients undergoing these surgeries. Due to the link between osteoporosis and mechanical issues, a holistic strategy for managing osteoporosis could significantly enhance surgical results. This inclusive approach would encompass medication usage, as well as lifestyle adjustments and nutritional assistance focused on improving bone strength.

The research underscores the significance of heightened surveillance and post-operative care, especially for high-risk patients. The early detection and management of complications can enhance results and decrease the need for more complicated revision surgeries. This study also stresses the importance of continuous training and education for surgeons to refine their surgical skills, as well as make knowledgeable choices regarding implant selection and surgical methods, enabling them to proficiently handle the complexities associated with intramedullary nailing.

### 4.5. Implications for Research

This research indicates various opportunities for future investigation. It is necessary to conduct comparative studies to evaluate diverse implant designs and surgical methods, aiming to identify those that reduce complications and improve patient outcomes. Furthermore, there is a distinct requirement for long-term studies assessing the effects of osteoporosis treatment on complication rates and patient recovery. Such studies could offer valuable insights for establishing evidence-based protocols for managing osteoporosis in relation to proximal femur fractures.

The creation of risk assessment methods that integrate demographic, intraoperative, and radiological elements has the potential to transform preoperative preparation. This may facilitate individualized patient treatment and well-informed clinical decision making. Furthermore, exploration into novel materials and technologies for intramedullary nailing can propel the field forward by possibly producing implants with enhanced biomechanical characteristics and lower failure rates.

Understanding the long-term outcomes and quality of life of patients who experience mechanical complications is critically important for advancing surgical techniques and rehabilitation protocols. It can ultimately improve patient care and treatment outcomes in proximal femur fractures by providing valuable insights into these crucial aspects. Expanding our understanding of these outcomes is essential for driving progress in this field.

## 5. Conclusions

Our findings suggest an association between osteoporosis treatment and a reduced incidence of mechanical complications. However, this may also reflect broader health management behaviors among these patients. Further research is needed to delineate the effects of osteoporosis treatment from other confounding factors. 

Our observations on risk factors in complication rates highlight the need for personalized approaches to minimize complications in diverse patient groups. More research is needed on the role that augmentation can play to avoid them, as well as comparing the results with other implants.

By considering these clinical and research implications, the orthopedic clinicians can progress in enhancing the treatment of proximal femur fractures, decreasing the occurrence of mechanical issues, thus improving the general health of their patients.

## Figures and Tables

**Table 1:** Baseline demographics.

Mean age	80
Sex	
Female	96
Male	30
Tobacco	
Smoker	21
Non smoker	105
Hypertension	
Yes	95
No	31
Diabetes mellitus	
Yes	42
No	84
Dyslipidemia	
Yes	74
No	52
Osteoporosis treatment	
Yes	28
No	98
Functional life (daily activities)	
Independent	94
Partially dependent	13
Dependent	19
ASA	
1	1
2	66
3	52
4	7
AO Fracture classification	
31.A1	31
31.A2	72
31.A3	23
Side	
Right	71
Left	55

Legend: ASA: American Anesthesia Association Classification; AO: Arbeitsgemeinschaft für Osteosynthesefragen system.

**Table 2:** Radiological measurements.

	*n* = 126	
Medial cortex contact AP view (frequency and percentage)	Negative	22 (16.1%)
	Neutral/Positive	104 (83.9)
Rupture of the lateral cortex AP view (frequency and percentage)	No	79 (64.5%)
	Yes	47 (35.5%)
CHANG (frequency and percentage)	1	5 (40)
	2	75 (59.7)
	3	46 (36.3)
Blade position acc to Cleveland (frequency and percentage)	Center	67 (61.3)
	Superior	27 (38.7%)
	Inferior	32
Intraoperative TAD average (mm)		18

Legend: AP: antero-posterior view; TAD: tip–apex distance; mm: millimeter.

**Table 3:** Mechanical Complications.

	*n* = 126	
Mechanical complications (frequency and percentage)	No	88 (79%)
	Yes	27 (21%)
Type of complication (frequency and percentage)	Breakage	4 (3.2%)
	Cut-out	8 (6.4%)
	Back-out	22 (17.4%)
	Cut-through	4 (3.2%)

Legend: *n*: number.

## Data Availability

Data are contained within the article.
